# Molecular approaches to determine the multiplicity of *Plasmodium* infections

**DOI:** 10.1186/s12936-018-2322-5

**Published:** 2018-04-23

**Authors:** Daibin Zhong, Cristian Koepfli, Liwang Cui, Guiyun Yan

**Affiliations:** 1 Program in Public Health, University of California, Irvine, CA 92617 USA; 2 Department of Entomology, Pennsylvania State University, University Park, PA 16802 USA

**Keywords:** Malaria parasite, Multiplicity of infection, Molecular marker, Next generation sequencing, Amplicon ultra-deep sequencing, Single nucleotide polymorphism

## Abstract

Multiplicity of infection (MOI), also termed complexity of infection (COI), is defined as the number of genetically distinct parasite strains co-infecting a single host, which is an important indicator of malaria epidemiology. PCR-based genotyping often underestimates MOI. Next generation sequencing technologies provide much more accurate and genome-wide characterization of polyclonal infections. However, complete haplotype characterization of multiclonal infections remains a challenge due to PCR artifacts and sequencing errors, and requires efficient computational tools. In this review, the advantages and limitations of current molecular approaches to determine multiplicity of malaria parasite infection are discussed.

## Background

Individuals in malaria-endemic countries often carry several genetically distinct clones of the same parasite species. Multiplicity of infection (MOI), also termed complexity of infection (COI) is defined as the number of genetically distinct parasite strains co-infecting a single host [1]. Multiclonal infection can be the result of independent bites of infected mosquitoes (also called superinfection), or a single mosquito bite transmitting a genetically diverse sporozoite inoculum. Genetically-distinct malaria parasites in natural populations have an extremely high rate of genetic recombination during the sexual stages in a mosquito host, often resulting in multiple strains being transmitted simultaneously [2]. In the case of *Plasmodium vivax*, multiclonal infections also can be caused by the relapse of a liver-stage hypnozoite during ongoing blood-stage infection of a genetically distinct clone. The number of co-infections within a host might be an important indicator of transmission intensity, though data to date are inconclusive [3–9]. For example, a positive correlation between the rate of polyclonal infections and annual parasite incidence has been observed in parasite populations from Indonesia [5] and Papua New Guinea [3], while in other studies, no correlation or negative correlation was found between the proportion of multiclonal infections and parasite prevalence [3, 6]. Several studies reported correlations between clinical symptoms and higher MOI [10–18] and others did not find any associations [19–21]. On the contrary, some studies reported that a reduced risk of clinical malaria was associated with polyclonal infections [22–24], while other studies reported that mono-infections and very common genotypes are more likely to develop severe malaria than polyclonal infections [21, 25]. MOI can also help to monitor vaccine efficacy. Vaccine-induced immune responses often show strain-transcending specificity [26, 27], depending on the polymorphic alleles of the vaccine candidate antigens [28]. Neafsey et al. found that the RTS, S vaccine has greater activity against malaria parasites with matched circumsporozoite protein allele than against mismatched parasite strains [29]. Hence, a reduction in MOI might be observed after vaccination, even if the risk of infection does not change. Understanding of the extent and dynamics of genetic diversity in vaccine antigens of all parasite strains is needed to guide rational vaccine design and to interpret the results of vaccine efficacy trials conducted in malaria endemic areas [30]. In drug trials, detecting all clones is important, as only one of them might be drug resistant and will result in recrudescence. If this clone is not detected at baseline, e.g., because it is present in a very low proportion of all clones, genotyping might falsely identify it as new infection and thus underestimate treatment failure. The molecular force of blood-stage infection (molFOB), i.e. the number of clones acquired over time, is an important parameter to estimate exposure at the individual level [31, 32]. Accurate estimates of molFOB require identification of all clones in multiclonal infections. However, sensitive identification of the within-host parasite component in multiclonal infection is often difficult due to lack of appropriate methods and analysis tools. In this review, the advantages and limitations of current molecular approaches to determine multiplicity of malaria parasite infection are assessed.

## Size-polymorphic antigens and microsatellites

Over the past decade, hundreds of papers describing the multiplicity of *Plasmodium* infections have been published by typing size-polymorphic antigens or microsatellite markers [18, 21, 28, 33–45]. Size-polymorphic antigenic markers, such as *msp*-*1*, *msp*-*2*, *msp*-*3*, *glurp*, *csp* and *ama*-*1* in *Plasmodium falciparum* and *P. vivax*, can be amplified by PCR, and the size of the amplicon is determined by either gel or capillary electrophoresis. Protocols are comparably simple without requiring expensive equipment. In many populations, high diversity of size-polymorphic antigens has been observed in both *P. falciparum* [33, 34, 38, 43, 46–53] and *P. vivax* [54–63]. Antigens are likely under immune selection, which will affect the distribution of alleles in the population. While in most cases this will not affect estimates of MOI, antigen markers should not be included in studies assessing parasite population structure.

Microsatellite markers are highly polymorphic with tandem repeats of 2–6 bp. They are expected to be selection neutral with no phenotypic consequences [64–67]. Microsatellites are widely distributed throughout the genome and can be readily amplified by PCR [68]. A range of 5–14 microsatellite markers have been used to estimate MOI in *P. falciparum* [53, 69–71], *P. vivax* [11, 72–74], and *Plasmodium malariae* [75, 76]. After evaluating 42 microsatellite markers in *P. vivax* from 18 different global studies, Sutton et al. found that five (*3.27*, *8.504*, *PvMS11*, *MS16*, and *MS8*) markers could be used as candidates for MOI studies [77]. However, as stutter peaks and nonspecific amplification in the case of suboptimal PCR conditions have been frequently observed [78–83], the recommendations of markers also need to take into account the robustness of the PCR protocols. High levels of stutter peaks have been observed in different laboratories for markers Pv3.27, MS8, and MS16 (Koepfli, unpublished), thus these markers are no longer recommended. In *P. falciparum*, there are 12 microsatellite markers (*Polyα*, *TA42*, *TA81*, *TA1*, *TA109*, *TA87*, *TA40*, *2490*, *ARAII*, *pfG377*, *PfPk2* and *TA60*) from 7 chromosomes that are frequently used for genetic diversity and multiplicity of infection studies [35, 65, 84]. MOI is determined as number of alleles detected, and if several markers are combined, as the maximum number observed for any marker. Any highly polymorphic marker is suitable for estimating the MOI.

The major limitation of agarose gel electrophoresis is its limited discrimination of alleles of similar sizes. It is unable to discriminate allele size difference less than 20 bp [85], resulting in underestimates of diversity and multiplicity. This problem is more pronounced when using microsatellites with many alleles differing by as little as 2–6 bp, as compared to antigens, that often harbor larger repeat sizes. Also, it is difficult to detect minority clones that result in weak bands on gels. Polyacrylamide gels have a better discrimination power. Even better results are obtained by using primers labeled with fluorophores and sizing the PCR products by capillary electrophoresis, which can resolve sizes of 2 bp. The second major problem is that PCR preferentially amplifies shorter fragments [86], and artifacts, such as chimera, emerge during multi-template PCR [87]. Furthermore, irrespective of markers used, PCR can lead to amplification from nonspecific template and produce PCR artifacts differing in length from the main products [80]. To avoid non-specific amplification due to mispriming and/or undesirable interactions between primers, the PCR conditions should be more stringent such as lowest possible MgCl_2_ concentration and the minimum number of amplification cycles.

## High-throughput single nucleotide polymorphism (SNP) genotyping

As alternatives to size-polymorphic markers, several panels of genome-wide SNP makers have been identified and protocols for typing developed. One panel includes 24 *P. falciparum* SNPs selected from over 112,000 SNPs of 18 parasite genomes [88]. Typing is based on High Resolution Melting (HRM) assays, which can detect parasite with a minor allele frequency higher than 10–20%, similar to that of traditional length polymorphic markers *msp*-*1* and *msp*-*2* [86], but the SNP-based molecular barcode provides greater power to discriminate among strains than *msp*-*1* and *msp*-*2* genotyping because many more possible alleles exist among the 24 markers than length polymorphisms within these regions. In *P. vivax*, a panel of 42-SNP barcodes was developed based on clinical samples from parasite populations in South America (Brazil, French Guiana), Africa (Ethiopia) and Asia (Sri Lanka) [89], and typed also using HRM assays. The small standardized set or subset of the 42-SNP barcode not only can be used for analyses of genetic diversity and population differentiation, but also can be used for MOI analyses though calculation of minor allele frequency [90]. HRM genotyping analyses provide a robust, informative, and relatively low-cost method of identifying parasite infections. However, a large amount of template DNA is required, as typically a separate reaction for each SNP needs to be run, and typing of low-density infections (e.g. from asymptomatic carriers) is challenging.

High-throughput SNP genotyping can also be done on the Sequenom MassARRAY iPLEX platform, which is capable of multiplexing up to 40 SNPs per single reaction. SNP allele is determined by variations of MALDI-TOF mass spectrometry at SNP site [91]. The parasite genotype is determined by the SNP allelic intensity ratios, ranging between 0 and 1. The SNP allelic intensity ratios value nearing 0 and 1 indicate single parasite genotype infection, while intermediate values indicate mixed parasite genotype infections [92]. Galinsky et al. described a method for estimating MOI based on the data obtained from Sequenom platform using likelihood and developed the software package COIL as implementation [93]. COIL assumes that distinct parasite lineages in complex infections are unrelated and that genotyped loci do not exhibit significant linkage disequilibrium, which is very suitable to employ SNP barcodes for MOI estimation. Similarly, the THE REAL McCOIL package was developed, which does not require external allele frequency data [94]. However, although this approach can be used for inferring the number of strains (COIL), or simultaneously inferring the number of strains and their proportions, it does not infer haplotypes.

Large-scale SNP genotyping using a custom 384-SNP Illumina GoldenGate has been conducted in *P. falciparum* from laboratory strains and clinical samples from East and West Africa, Southeast Asia, and Oceania [95]. This genotyping assay has a high genotype calling specificity from artificial mixtures of laboratory clones with a moderate sensitivity to call minor frequency alleles. The advantage of these methods is the large number of SNPs typed simultaneously. It thus can provide a practical, rapid, robust, and inexpensive assay of genome-wide parasite genotyping with easy data interpretation. The GoldenGate platform relies on monoclonal infections to estimate allele frequencies, which is problematic when a large fraction of infections are polyclonal. While most SNPs are bi-allelic, in theory up to four alleles are possible. Effective computational tools and advanced bioinformatic analysis are required to distinguish more than two parasite clones within a host [94].

Nkhoma et al. used a set of 96 genome-wide SNPs and Illumina GoldenGate to examine changes in parasite population genetic parameters associated with decline in transmission in *P. falciparum* [96]. Polyclonal infection was determined by heterozygous base calls at > 5% of the genotyped SNPs with an error rate of 4.13 × 10^−4^. The clonal composition of parasite populations was measured by genotypic richness index (*R*) and the β-parameter of the Pareto distribution. *R* measures the proportion of unique genotypes (*G*) in the population and is estimated as: *R* = (*G* − 1)/(*n* − 1), which varies from 0 when all *n* samples in a population have the same genotype, to 1.0, when all samples have a different genotype [97]. β-Parameter measures the slope of Power law (Pareto) distribution by resampling different numbers of samples (e.g. n = 100–1100) from the complete data set and recalculating genotypic data. The slope is highly skewed with a large number of rare genotypes and a few common ones [98]. A minimum of 25 SNP markers are required to exhaustively identify all distinct multilocus parasite genotypes present in infections [96]. However, the proportion of multiple-genotype infections is dependent on the sensitivity of genotyping methods used and is therefore difficult to compare between studies. Genotype richness and the β-parameter of the Pareto distribution can only be measured using single genotype infections.

## Amplicon ultra-deep sequencing

With the advent of next-generation sequencing (NGS) technology, studies on population genetics, diversity, and multiplicity of *Plasmodium* infections have entered a new era. NGS is becoming the standard approach to generate population genomic data. Great progress has been made for NGS data analysis and MOI estimation with the development of various bioinformatic tools (Table 1). In highly polymorphic amplicons, several SNPs are concentrated within a locus of 100–200 bp. Amplicon ultra-deep sequencing of high polymorphic makers has high sensitivity and specificity to detect minority clones in multiclonal infections. A number of highly polymorphic amplicons have been identified [99, 100]. Using *pvmsp1* short amplicon (117 bp) deep sequencing, Lin et al. reported 67 unique haplotypes identified from 78 Cambodian *P. vivax* samples with an average of 3.6 MOI within each individual [100]. To increase the sensitivity of detecting minor alleles, three *P. falciparum* amplicons of 321 bp (*pf*-*csp*), 305 bp (*pf*-*ama1*), and 306 bp (*pf*-*k13*) have been used to conduct ultra-deep sequencing, which can quantitatively detect unique haplotypes comprising as little as 2% of a polyclonal infection [101].

**Table 1:** The advantages and limitations of bioinformatics tools current available for analysis of polyclonal infections and estimation of MOI

Software/approach	Implementation	Data source	Advantage	Limitation	References
*Fws*	Fws statistics	SNPs	Provide a summary statistic of relative inbreeding and correlation with MOI	Does not provide a direct estimate of COI	Manske et al. [108]
*estMOI*	Perl scripts	TDS	estimate MOI, SNP combinations and proportion in a specific genomic region	Partial genomic region, sufficient density of SNPs in an amplicon is required	Assefa et al. [107]
*COIL*	Perl scripts web interface	SNPs	Use genome-wide SNPs to infer the number of strains and their proportions	Does not infer haplotypes; sequence read counts are assumed to be unbiased and the SNPs are unlinked	Galinsky et al. [93]
*SeekDeep*	Perl scripts	TDS	No reference sequence required, direct estimation of amplicon haplotype cluster and frequency	Partial genomic region, threshold for haplotype cluster calls needs to be determined by empirical methods in each study	Hathaway et al. [104]
*HaplotypR*	R package	TDS	Direct estimation of haplotype cluster and frequency based quality filtering of sequencing reads	Partial genomic region, enough high polymorphism in the amplicon is required and unable to deal with indel of amplicon	Lerch et al. [102]
*pfmix*	R package	SNPs	Infer inbreeding coefficients using WGS to assess within-sample parasite infections	Does not infer haplotypes, sequence read counts are assumed to be unbiased and SNPs need to be unlinked	O’Brien et al. [110]
*THE REAL McCOIL*	Perl scripts web interface	SNPs	Infer the number of strains and their proportions	Does not infer haplotypes, sequence read counts are assumed to be unbiased and SNPs need to be unlinked	Chang et al. [94]
*DEploid*	R package	SNPs	Estimate the number of strains, their relative proportions, and the haplotypes	Reference panel required, more reference strains are better. Multiallelic variant not considered	Zhu et al. [111]

*SNPs* single nucleotide polymorphism, *TDS* targeted deep sequencing, *WGS* whole genome sequencing, *MOI* multiplicity of infection

Recently, the conserved *Plasmodium* membrane protein (*cpmp*) was described as a highly polymorphic marker, and an assay was developed with 100% sensitivity to detect all clones present at a frequency of > 1% [102]. Ultra-deep sequencing of amplicons from ribosomal genes and genes encoded by the mitochondrial and apicoplast genomes has been used to evaluate *Plasmodium* species diversity and MOI in symptomatic Gabonese patients [103]. It is, however, challenging to distinguish PCR and sequencing errors from true minority haplotypes. Recently, multiple packages have been developed for de novo clustering of haplotypes from this type of data allowing detection of low frequency variants, such as HaplotypR [102] and SeekDeep [104].

Several studies have evaluated long amplicon deep sequencing to examine multiplicity of infection by sequencing mixtures of multiple lab strains. For example, Patel et al. performed amplicon deep sequencing of 1.6 kb ID1-DBL2× region of *var2csa* gene in *P. falciparum* infected placental samples and found that parasite haplotypes can be recovered if they account for as little as 5% of the mixed template [105]. A long amplicon (~ 5 kb) of *var2csa* gene in *P. falciparum* was sequenced by PacBio deep sequencing and used to evaluate artificially created a mixture of laboratory strains [106]. De novo assembly of full-length amplicons was used to reconstruct *var2csa* haplotypes. It however identified minor alleles only if present at frequencies > 23% in the mixture, due to low read depth and coverage and high sequencing errors in homopolymer stretches [106]. In order to obtain highly accurate haplotype estimation from polyclonal clinical samples, Lerch et al. suggest that a minimum coverage of 10,000 high-quality sequencing reads with duplicate experiment is required to detect minority clones at 0.1% frequency [102]. The major advantage of single amplicon deep sequencing over a panel of genome-wide SNPs is that all SNPs occur within one amplicon. Thus, haplotypes can be directly identified without the need for multi-locus haplotype reconstruction. Software such as SeekDeep aids in the identification of haplotypes [100, 104]. After filtering of sequencing reads according to their base quality scores, they are clustered using a clustering algorithm based on k-mer distances. In contrast to microsatellites and SNP-panels, the haplotype frequency can be directly estimated from read counts with high accuracy. The main limitation is that only a small genomic region is amplified. The threshold for accurate genotype calls may be different between studies due to various sequencing error rate in different sequencing platforms and computational strategies. In order to exclude PCR or sequencing errors, it is recommended to perform experiments in duplicate and use appropriate controls with each study to help determine that no false calls are being made.

## Whole genome sequencing

Whole genome sequencing of field isolates is rapidly becoming common, and a number of software packages have been developed to identify multiclonal infections and reconstruct haplotypes. The estMOI software package can be used to analyse deep sequencing data for estimation of the presence of multiple genotypes, the number of infections, as well as proportions by phasing information of each allele [107]. In other words, the software can be used to estimate minor allele (> 10% frequency) in multiclonal infections. The limitation of this software is that MOI is estimated by maximum number of haplotype in a specific genome region with a high coverage (> 30-fold), which is costly to generate at large scales by whole genome sequencing. In order to reduce cost, a list of 26 MOI informative genes with high levels of polymorphism has been recommended to use for genotyping to estimate multiplicity in clinical *P. falciparum* samples [107].

The F_WS_ (similar to Wright’s inbreeding coefficient F_IS_) statistics derived from read count data of whole genome sequencing at 86,158 SNP positions had been used to explore the number of within-host clones, their relative proportions and genetic divergence in *P. falciparum* [108, 109]. This approach is conducted by estimating levels of heterozygosity both within an individual sample (Hw) and within the local parasite population (Hs) using genome-wide SNP data. The F_WS_ values, where F_WS_ = 1 − H_W_ / H_S_, observed in blood samples provide a proxy indicator of inbreeding rates in the parasite population. The F_WS_ ranges from 0 to 1, with 0 reflecting a mixture of highly unrelated clones and 1 reflecting a single clone. In other words, a low F_WS_ reflects a low risk of inbreeding/high risk of out-crossing and thus high within-host diversity. A threshold of F_WS_ ≥ 0.95 was suggested as indication of an essentially clonal infection. A significant negative correlation was observed between the PCR-based *msp1* genotyping and F_WS_ statistics for calculating MOI. Although the F_WS_ can capture information of within-host heterozygosity relative to population diversity, it does not provide a direct estimate of MOI. To help make a connection of F_WS_ with MOI estimates, O’Brien et al. presented several new approaches for inferring inbreeding coefficients using read counts from WGS, which can be implemented in the R package [110].

Zhu et al. developed a software package named *DEploid*, which can be used to analyse genome-wide SNP data for estimation of the number of strains, their relative proportions and the haplotypes present in a sample [111]. The software package uses haplotype structure within a reference panel of clonal isolates as a prior for haplotypes present in a multiclonal sample. The effective number of strains can be inferred without an assumption of the number of strains or the use of linkage disequilibrium information. The assumed haplotypes are inferred using read counts of the reference strain and alternative alleles. First, allele counts are extracted from the vcf file, and then the population allele frequencies of each allele are calculated from total read counts. Finally, inference of correct haplotypes and relative proportions can be completed by quality filtering of sequencing reads. Overall, this method can be used to infer mixed strains with proportions at > 20% with high accuracy, but it struggles with minor strains due to insufficient read coverage and if the minor strain carries the alternative allele. The computer program has been tested and confirmed to work well for the deconvolution of datasets with a mixture of samples from a single species in *P. falciparum* [111]. It is able to perform deconvolution of 372,884 variant sites of malaria parasites within one sample in just five and half hours. *DEploid* is the only software currently available that can reconstruct haplotypes as well as estimate COI using genome wide SNP data. The limitation is that it requires a panel of references, which should include enough different reference strains to cover all the haplotype structure representing the field population. However, this is not realistic for current computational technology.

Finally, sequencing coverage remains a main challenge when estimating MOI from whole genome sequencing data. By amplicon deep sequencing, a read depth of 1000–10,000× coverage is easily obtained, resulting in a significantly increased power of detection of minority clones. At this sequencing depth, minority clones at a frequency of 0.1% have been successfully identified in experimental mixtures of strains [102]. A typical sequencing run on an Illumina NextSeq sequencer yields approximately 130 million reads of 150 base pairs each. Even if a single isolate was sequenced by a single run, and if even coverage across the genome of 23 million base pairs was obtained, coverage would be below 900×. Factoring in that field isolates always contain some human DNA, coverage would be even lower. Thus, when MOI is the main interest of a study, whole genome sequencing is not a cost-effective method at the current stage.

## Conclusions

Traditional standard PCR-based genotyping methods using *msp*-*1, msp*-*2*, and microsatellite markers often underestimate the MOI. Next-generation amplicon sequencing technology has largely increased the sensitivity in detection of polyclonal infections, but targets only partial genomic regions, which may not represent complete polymorphism in mixed infections. Whole genome sequencing yields complete information for parasite infections, but requires extensive and efficient computation algorithms and mathematical models to reconstruct the complete haplotypes, and sensitivity to detect minority clones is limited due to low reads coverage for minor strains. Therefore, there is a need to develop an accurate and cost-effective method for detection of minority clones at the current stage. As the cost of whole genome sequencing continues to decrease and bioinformatics tools mature, fine-scale investigation of multiplicity of Plasmodium infections becomes possible in the near future.

## Abbreviations

COI: complexity of infection
COIL: complexity of infection using likelihood
HRM: high resolution melting
LOD: limit of detection for minority haplotype
MOI: multiplicity of infection
SNPs: single nucleotide polymorphism
TDS: targeted deep sequencing
WGS: whole genome sequencing
